# *BRCA* testing patterns in breast cancer over time in the United States: challenges and opportunities for improvement

**DOI:** 10.3389/fonc.2026.1797497

**Published:** 2026-04-27

**Authors:** Kathryn Mishkin, Erin Mandal, Deborah Tallarigo, Qixin Li, Carolina Casañas i Comabella, Michael Byrnes, Kelly E. McCann, Laura Moreno, Diane Rose

**Affiliations:** 1 Merck & Co., Inc., Rahway, NJ, United States; 2 Thermo Fisher Scientific, Waltham, MA, United States; 3 Thermo Fisher Scientific, Montreal, QC, Canada; 4 AstraZeneca, Gaithersburg, MD, United States; 5 Thermo Fisher Scientific, London, United Kingdom; 6 Division of Hematology/Oncology, David Geffen School of Medicine at the University of California, Los Angeles, Los Angeles, CA, United States; 7 Clinical Cancer Genomics, City of Hope, Atlanta, GA, United States; 8 Facing Our Risk of Cancer Empowered (FORCE), Tampa, FL, United States

**Keywords:** BRCA mutations, breast cancer, germline testing, olaparib, PARP inhibitors

## Abstract

Testing for germline mutations in breast cancer genes (g*BRCA*m) is recommended to inform treatment decisions for patients with breast cancer (BC); however, not all eligible patients will undergo testing. This targeted literature review summarizes evidence on real-world patterns of g*BRCA*m testing in BC in the United States (US) and misconceptions, beliefs, attitudes, or barriers related to g*BRCA*m testing. A total of 35 publications published up to September 2024 were included in the review, representing 32 unique studies. Since the early 2000’s, studies consistently reported increases in *BRCA* testing over time (7%-47% increase, with variations based on differing study designs, populations, and assessment periods). However, an unmet need for improved testing remains, particularly for eligible patients with hormone receptor-positive early BC. Barriers to testing reported by patients and physicians include lack of clear guidelines around g*BRCA*m testing eligibility, high costs, and inadequate insurance coverage. Additionally, patients with BC often reported limited knowledge regarding g*BRCA*m testing. Variable access to genetic counseling was reported by physicians as both a driver and a barrier to testing, and some physicians lack the expertise or confidence to discuss testing results with patients. Further evaluation on recent g*BRCA*m testing trends and barriers related to *BRCA* testing in the US is needed as the majority of studies identified in this literature review had data collection periods that occurred prior to 2020.

## 1 Introduction

Mutations in breast cancer genes 1 (*BRCA1*) and 2 (*BRCA2*) were first found to be associated with breast cancer (BC) risk in the mid-1990s (1). *BRCA1* and/or *BRCA2* mutations can occur as germline mutations (g*BRCA*m), which are inherited and occur in all cells throughout the body, and somatic mutations, which are acquired mutations only present in tumor cells (2, 3). Approximately 55% to 72% of g*BRCA1* mutation carriers and 45% to 69% of g*BRCA2* mutation carriers assigned female at birth will develop BC in their lifetime, compared to 12% among the general population (4).

g*BRCA*m are associated with breast cancer diagnosis at an early age, the development of higher grade tumors, and triple-negative BC (TNBC), which has higher rates of recurrence and mortality compared to other BC subtypes (5–9). Patients with *BRCA1* mutations have an increased risk of TNBC, whereas the majority of patients with *BRCA2* mutations have hormone receptor positive (HR+)/human epidermal growth factor receptor 2 (HER2)-negative disease (10, 11). Although g*BRCA*m are associated with diagnosis at an earlier age and TNBC, more than half of all patients with BC in the US are diagnosed at ≥60 years of age, and HR+/HER2- disease is the most common BC subtype (12, 13). Accordingly, the majority of patients with g*BRCA*m breast cancer have HR+/HER2-negative disease and are aged >50 years, suggesting an unmet need for g*BRCA*m identification in this population (14–17).

According to National Comprehensive Cancer Network (NCCN) Clinical Practice Guidelines in Oncology (NCCN Guidelines^®^) for Genetic/Familial High-Risk Assessment: Breast, Ovarian, Pancreatic, and Prostate. V.2.2026., genetic testing for high-penetrance BC susceptibility genes, including g*BRCA*m, is clinically indicated in patients with a personal history of BC if they are ≤50 years of age, or at any age in patients with BC who have specific demographic or clinical characteristics (18). These characteristics include (but are not limited to) family history of BC or other cancers suggestive of the possibility of the pathogenic variant, specific pathology/histology (e.g., TNBC), personal or family history of male breast cancer, and Ashkenazi Jewish ancestry (18). Testing and genetic counseling may also be considered among patients with a personal history of BC who are aged ≤65 years without specified demographic or clinical characteristics (18). The 2024 American Society of Clinical Oncology (ASCO)-Society of Surgical Oncology (SSO) guidelines recommend that g*BRCA*m testing be offered to all patients newly diagnosed with BC who are aged ≤65 years, or those aged >65 years with specific demographic or clinical characteristics (19). The American Society of Breast Surgeons (ASBrS) also recommend genetic testing for all patients with a personal history of breast cancer and for patients without a personal history of breast cancer according to NCCN Guidelines^®^ criteria (20).

Testing for g*BRCA*m among high risk individuals without a BC diagnosis can inform enhanced surveillance and risk management strategies (e.g., risk-reducing bilateral mastectomy) (18, 21). Among patients with BC, genetic testing for g*BRCA*m is clinically indicated and guideline-recommended to inform treatment decisions, including surgery and use of targeted therapy with poly(ADP-ribose) polymerase (PARP) inhibitors (3, 18, 19, 21). The PARP inhibitor talazoparib was approved in 2018 for the treatment of patients with g*BRCA*m, HER2-negative, locally advanced or metastatic BC based on significant improvements in PFS reported in the phase 3 EMBRACA trial (22, 23). The PARP inhibitor olaparib was approved in 2022 for the treatment of g*BRCA*m, HER2-negative, early stage BC (eBC) at high risk of recurrence based on the significant invasive disease-free survival, distant disease-free survival, and overall survival benefits reported in the phase 3 OlympiA trial (24, 25). With the approval of olaparib, timely genetic testing has become critical for the appropriate treatment of patients with eBC who carry g*BRCA*m. Conventional hormone therapies targeting the estrogen receptor (ER) and progesterone receptor (PR) and therapies targeting HER2 are largely ineffective in treating TNBC, with the exception of trastuzumab deruxtecan for the treatment of HER2-low disease (7, 26).

**Figure 1:**
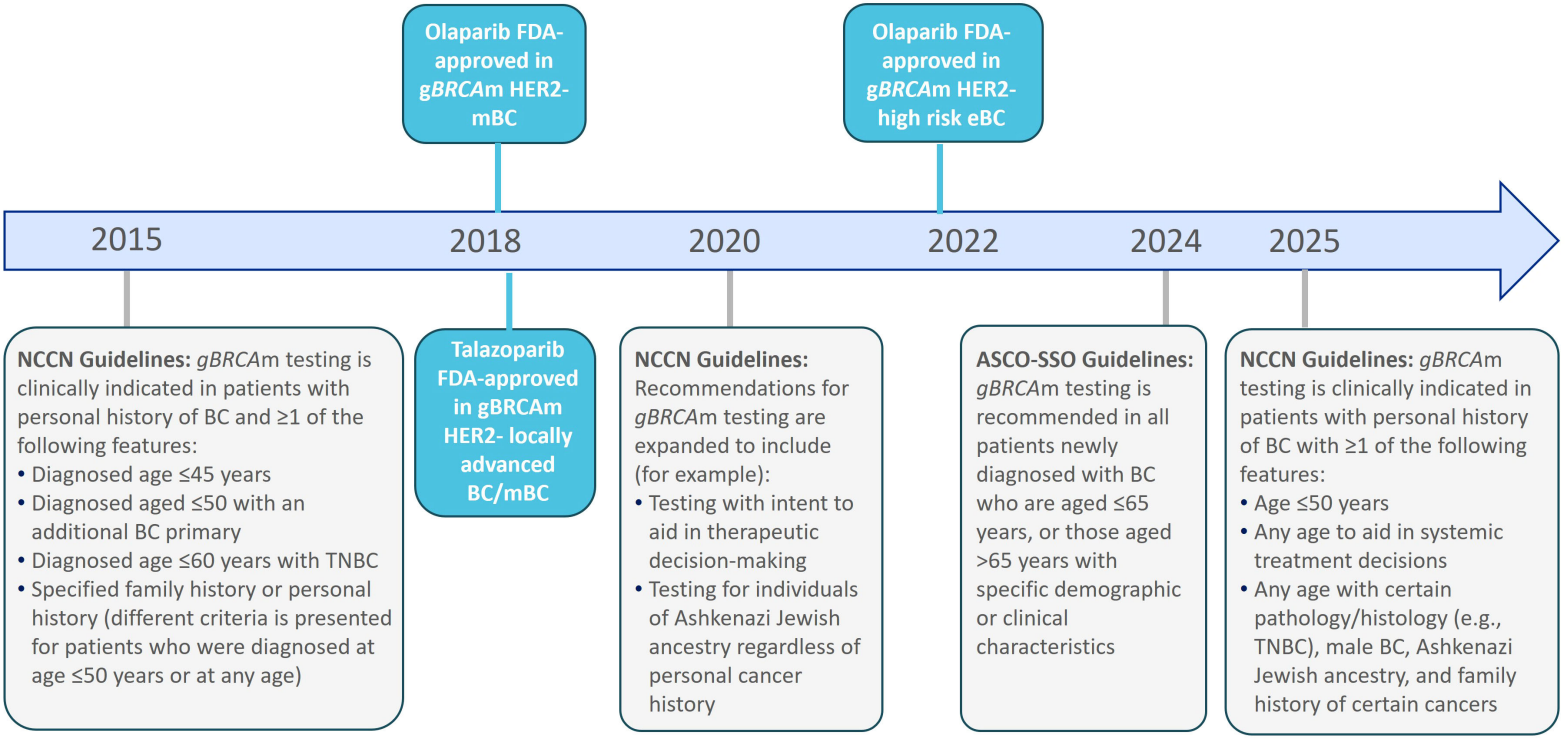
Overview of changes in recommendations for genetic testing and treatment approvals for g*BRCA*m breast cancer in the US. This figure illustrates a topline overview of selected recommendations for genetic testing in patients with BC who are considered at risk of having g*BRCA*m. It is not a comprehensive description of all genetic testing recommendations per the NCCN or ASCO-SSO, and it does not illustrate all changes observed in the NCCN Guidelines or ASCO-SSO recommendations from 2015 to 2025. Please refer to the NCCN Guidelines for more details. NCCN makes no warranties of any kind whatsoever regarding their content, use or application and disclaims any responsibility for their application or use in any way. ASCO-SSO, American Society of Clinical Oncology-Society of Surgical Oncology; BC, breast cancer; eBC, early breast cancer; FDA, Food and Drug Administration; gBRCAm, germline breast cancer gene mutation; HER2, human epidermal growth factor receptor 2; mBC, metastatic breast cancer; NCCN, National Comprehensive Cancer Network; TNBC, triple negative breast cancer; US, United States. Source: Illustration based on Bedrosian et al., 2024 (19), Daly et al., 2016 (27), Daly et al., 2020 (28), NCCN Guidelines V2.2026 (18), Olaparib Prescribing Information (24), and Talazoparib Prescribing Information (22).

As genetic testing criteria for patients with BC have expanded within the past decade (**Figure 1**), there has been an increased uptake of g*BRCA*m testing to guide treatment decision-making (18, 29). The costs of genetic testing have decreased over the same timeframe (30, 31). Median per-person expenditures related to g*BRCA*m testing for a privately-insured individual decreased 68% from 2013 to 2022, and evidence suggests out of pocket costs for germline testing is approximately $100 to $250 for a patient newly diagnosed with cancer (30, 32). However, despite guideline recommendations, the literature demonstrates a gap in g*BRCA*m testing among all eligible patients with BC (33, 34). The objective of this review was to identify and synthesize US-based scientific literature on the real-world patterns of g*BRCA*m testing in BC and misconceptions, beliefs, attitudes, or barriers related to g*BRCA*m testing.

## 2 Materials and methods

### 2.1 Data sources

Databases of published literature (Embase, MEDLINE, MEDLINE In Progress, and PsycINFO) were searched utilizing a combination of free-text searches and medical subject headings (MeSH) terms via the OvidSP^®^ platform on September 5, 2024 (**Supplementary Tables 1**, **2**). Manuscripts published any time and conference abstracts published from 2022 onwards were eligible for inclusion.

### 2.2 Study selection

We included US-based real-world studies (e.g., observational studies, qualitative studies, or survey studies) that evaluated patients diagnosed with BC (any stage) who were eligible for *BRCA* testing and/or healthcare professionals (HCPs) who are involved in *BRCA* testing decision making (e.g., oncologists, pathologists, surgeons, genetic counselors, nurses, and payers). Studies evaluating individuals eligible for *BRCA* testing who were not diagnosed with BC were not eligible for inclusion in this literature review. Studies containing information on the following topics were selected for inclusion:

- Real-world *BRCA* testing patterns, including the proportion of patients with BC receiving or refusing a *BRCA* test and the timing of *BRCA* testing.
- Misconceptions (e.g., beliefs regarding testing guidelines and procedures that are not supported by current evidence), barriers, beliefs, or attitudes about *BRCA* testing for treatment decisions, as reported by patients or healthcare professionals.

To prioritize inclusion of the most robust, recent evidence in our literature review, we excluded studies with a publication date prior to 2014 and studies reporting on real-world testing patterns with a sample size <450 patients.

### 2.3 Review procedure

Included studies were imported to Nested Knowledge^®^, an online systematic review platform where references identified from more than 1 database were removed as duplicates. Two levels of screening were completed based on the predetermined population, interventions and comparisons, outcomes, and study design (PICOS) selection criteria (**Supplementary Table 3**), including screening of titles and abstracts followed by full text screening. Artificial intelligence (AI) algorithms available in Nested Knowledge were used to expedite the screening of titles and abstracts. To reduce the volume to be screened, citations that had a low probability of inclusion based on the AI model (approximately ≤20% probability of inclusion) were screened only at the title level by a single, independent reviewer. All remaining titles and abstracts were screened by a single reviewer. A single independent reviewer completed full text screening (no AI was used at this stage). A second independent researcher performed standard quality checks on approximately 10% of randomly selected references during the abstract and full text screening to verify results. Data were extracted into an Excel-based data extraction template by a single reviewer, and results were verified by a second reviewer.

## 3 Results

A total of 2,003 records were screened; of these, 35 publications, representing 32 unique studies, were included (**Supplementary Figure 1**). Real-world *BRCA* testing patterns were described in 30 references, representing 28 unique studies. Beliefs, attitudes, and misconceptions related to *BRCA* testing in BC among patients, HCPs, and payers were described in 7 unique studies reported in 9 publications. Perceived barriers to *BRCA* testing in BC among different stakeholders (patients, HCPs, and payers) were described in 11 unique studies reported in 13 publications.

### 3.1 Real-world *BRCA* testing patterns

#### 3.1.1 *BRCA* testing rates

Real-world *BRCA* testing rates among patients diagnosed with BC ranged broadly in mixed samples of BC (approximately 14% to 87%). Since the early 2000’s, studies consistently reported increases in *BRCA* testing over time (7% to 47% increase, with variations based on differing study designs, populations, and assessment periods) (**Table 1**) (36–45). Studies had heterogenous data collection periods, patient inclusion and exclusion criteria (e.g., age or disease stage), and time frames in which *BRCA* testing was reported (e.g., within 6 months to 1 year of diagnosis, within 6 months of surgical treatment, or at any point during the entire study period). Nine studies reported increasing rates of testing over time (36, 37, 39–41, 43–46). Sixty four percent of HCPs routinely ordered g*BRCA*m testing based on a 2023 survey (115 oncology providers) compared with 29% of HCPs based on a 2018 survey (95 oncology providers) (46). For patients with eBC, 23% of providers routinely ordered g*BRCA*m testing in 2023, representing a threefold increase compared to 2018 (46).

**Table 1:** Real-world *BRCA* testing patterns in the US among patients with breast cancer.

Author, year	Study overview / data collection period	Patient population	Overview of RW BRCA testing rates	Key RW BRCA testing trends over time
Shao, 2024 (35)	Retrospective database study (Flatiron Health) 2018-2020	aBC; aged ≥18 years N=1,275	*BRCA testing before first-line therapy for aBC:* 13.5% of patients who received ≥1 systemic treatment	NR
Dinan, 2023 (36)	Retrospective database study (Blue Cross Blue Shield Axis) 2015-2019	Newly diagnosed, surgically treated invasive BC; aged ≥18 years N=137,535	*BRCA testing within 6 months of surgery:* 40%	*BRCA testing within 6 months of surgery:* 2015, H1: 38% 2019, H2: 47% Testing rates increased steadily over time (*p*<0.001, trend over time)
Lau-Min, 2023 (37)	Retrospective, database study (Flatiron Health) 2011-2020	BC diagnosed at ≤45 years of age or TNBC diagnosed at ≤60 years of age N=2,982	g*BRCA*m *testing during study period:* 56.4% tested within 1 year of diagnosis 7.1% tested 1 year after diagnosis	Rates of g*BRCA*m testing increased significantly over time (RR: 1.08 [95% CI: 1.05 to 1.11], for each year after 2011), independent of when PARPi were approved for g*BRCA*m mBC in January 2018 g*BRCA*m *testing within 1 year of diagnosis:* 2011: 37.0% 2020: 67.9%
Wang, 2023 (38)	Survey 2013-2015	BC; aged ≥18 years N=93	*BRCA testing prior to survey:* 34.4%	NR
Pace, 2022 (39)	Retrospective database study (Massachusetts' APCD and MCR) 2010-2013	BC; aged 18-45 years N=2,424	*BRCA testing within 6 months of diagnosis:* Stage 0: 42.6% Stage I: 58.3% Stage II: 60.2% Stage III: 58.7% Stage IV or unknown: 42.9%	*BRCA t*esting was significantly more likely for patients diagnosed in 2013 versus 2010 (OR: 1.45 [95% CI: 1.14 to 1.84])
Kurian, 2021 (40)	Retrospective study (SEER registries) 2013-2017	BC; aged ≥20 years N=187,535	g*BRCA*m *testing during study period:* 25.2%	Genetic testing rates for any germline mutations generally increased approximately 2% each year from 2013 through 2017
Stenehjem, 2021 (41)	Retrospective single-center study 1995-2014	BC; aged ≥18 years N=5,712	g*BRCA*m *testing during study period:* 14.6%	g*BRCA*m *testing by 5-year period* 2000-2004: 5.7% 2005-2009: 17.1% 2010-2014: 23.1%
Kurian, 2019 (42)	Retrospective study (SEER registries) 2013-2014	BC; diagnosed at ≥20 years of age N=77,085	g*BRCA*m *testing during study period:* Stage 0: 19.7% Stage I: 23.5% Stage II: 28.6% Stage III: 29.4% Stage IV: 16.2% Note: rates are for any genetic testing; >99% of all tested patients were tested for g*BRCA*m	NR
Kehl, 2016 (43)	Retrospective, database study (MarketScan) 2005-2012	BC diagnosed at ≤45 years of age N=26,985	*BRCA testing within 1 year of surgery, in 2012:* Patients aged ≤40 years: 72.9% Patients aged 41-45 years: 65.3%	Rates of *BRCA* testing generally increased over time for patients aged ≤40 years (26.0% in 2005; 73.0% in 2012) and patients aged 41-45 years (20.5% in 2005 and 64.8% in 2012), based on Kaplan-Meier analysis
Rosenberg, 2016 (44)	Prospective cohort study 2006-2014	BC, diagnosed at ≤40 years of age N=897	*BRCA testing by 1 year after diagnosis:* 87.0%	*BRCA testing by 1 year after diagnosis:* 2006: 77% 2013: 95%
Wright, 2016 (45)	Retrospective database study (MarketScan) 2009-2013	Newly diagnosed, surgically treated BC N=116,793 women N=874 men	*BRCA testing within 6 months of diagnosis:* Women: 22.9% Men: 31.0%	*BRCA testing within 6 months of diagnosis:* 2009, women: 20.0% 2013, women: 26.5% (*p*<0.001 vs 2009) 2009, men: 20.9% 2013, men: 41.6% (*p*<0.001 vs 2009)

aBC, advanced breast cancer; APCD, All Payers Claims Data; BC, breast cancer; BRCA, breast cancer gene; eBC, early breast cancer; gBRCAm, germline BRCA mutation; HER2, human epidermal growth factor receptor 2; H1, first half; H2, second half; HR, hormone receptor; MCR, Massachusetts Cander registry; NR, not reported; OR, odds ratio; PARPi, PARP inhibitors; RR, risk ratio; RW, real-world; TNBC, triple negative breast cancer; US, United States.

Variations in *BRCA* testing rates were observed according to BC subtype. Among 4 studies that evaluated testing rates among patients with advanced or metastatic BC, higher rates of g*BRCA*m testing were reported for patients with TNBC (ranging from 24% to 73%) compared to those with HR+/HER2-negative BC (ranging from 15% to 43%); however, these studies did not report information on the types of tests used (e.g., next generation sequencing) (17, 47–49). Conversely, in 1 study evaluating patients with eBC, the rate of single or multi-gene *BRCA* testing for TNBC (16%) was lower compared to patients with HR+/HER2-negative eBC (25%) (50). *BRCA* testing rates in HR+/HER2-negative eBC ranged from 25% to 32% in 2 studies evaluating patients regardless of recurrence risk status, with evidence demonstrating that g*BRCA*m testing rates within 6 months of diagnosis increased from 14% during 2011 to 2014 to 32% from 2019 to 2022 (50, 51).

Patients considered at high risk of carrying a *BRCA* mutation consistently had higher testing rates than patients with low or moderate risk (**Table 2**) (52–56). The criteria used to define risk of carrying a *BRCA* mutation was based on the NCCN Guidelines in all 5 studies identified, and the definition of “high risk” was expanded in studies that referred to more recently published versions of the NCCN Guidelines (e.g., 2017) compared to older versions (e.g., 2007) (52–56). One retrospective chart review study using NCCN Guidelines published from 2013 to 2017 found that most (94.4%) patients with eBC and a high risk of having a g*BRCA*m ultimately received *BRCA* testing (53). A survey published in 2023 evaluated the rates of g*BRCA*m testing among patients with HR+/HER2-negative eBC who were considered at high risk of disease recurrence and therefore eligible for g*BRCA*m testing (57). In this study, medical oncologists (n=94), surgeons (n=97) and nurses and physician assistants (n=58) reported that only approximately two-thirds of these patients received g*BRCA*m testing (57). No consistent definition of high risk of disease recurrence was used in this study, and survey participants reported using several different educational resources to define high risk, such as NCCN Guidelines, tumor boards, and peer-reviewed journals (57).

**Table 2:** Real-world *BRCA* testing patterns in the US among patients with breast cancer based on risk.

Author, year	Study overview/ data collection period	Patient population	Key results
Testing rates based on risk of having *BRCA* mutation			
Law, 2022 (52)	Retrospective database analysis (Syapse LHN) Risk of *BRCA* mutation per NCCN Guidelines published in January 2015 2015-2020	Patients diagnosed with TNBC at ≤60 years of age N=577	Among 100% of patients who met NCCN Guidelines recommendations for testing, 79.5% received testing
Bobbili, 2020 (53)	Retrospective chart review Risk of *BRCA* mutation per NCCN Guidelines, as available from 2013 to 2017, i.e., the years that enrolled patients were diagnosed with BC) 2017-2018	BC with high risk of having *BRCA* mutation N=410	93.7% of high risk patients received testing Patients tested in each risk group: Men with a personal history BC: 90.0% Women with ≥1 close blood relative with a *BRCA* pathogenic variant: 100% Women diagnosed with BC at ≤45 years of age: 94.0% Women diagnosed at ≤50 years of age with: An additional primary BC: 100% ≥1 close blood relative with BC: 94.8% ≥1 close blood relative with prostate cancer (Gleason score ≥ 7): 86.8% Women diagnosed at ≤60 years of age with TNBC: 96.1% Women with a personal history of ovarian cancer: 100% Women diagnosed at any age with: ≥1 close blood relative with BC diagnosed at age ≤ 50 years: 93.9% ≥2 close blood relatives on the same side of the family with BC: 92.7% ≥1 close blood relative with ovarian cancer: 93.1% ≥2 close blood relatives on the same side of the family with pancreatic and/or prostate cancer (Gleason score ≥ 7): 91.5% ≥1 close male blood relative with BC: 77.8% Ashkenazi Jewish or ethnic groups associated with founder mutations: 97.3%
Pederson, 2018 (54)	Retrospective single center study Risk of g*BRCA*m per NCCN Guidelines, version 1.2018) 2013-2016	TNBC N=477	Among 69.4% of patients who met NCCN Guidelines recommendations for genetic testing, 68.3% received testing
Jones, 2017 (55)	Survey Risk of *BRCA* mutation per NCCN Guidelines, version 1.2007 2007-2009	Black women aged 18–64 years with eBC N=945	Among all women who were tested (n=252): 49.2% were high risk 34.9% were moderate risk 15.9% were low risk Among women who were not tested (n=693): 22.2% were high risk 39.5% were moderate risk 38.2% were low risk
Kishan, 2016 (56)	Retrospective single center study Risk of *BRCA* mutation per (NCCN Guidelines, version 2.2016) 2012-2015	Non-metastatic BC N=609	Among 51.3% patients who met NCCN Guidelines recommendations for testing, 88.8% were referred for testing Of referred patients, 89.9% received testing
Testing rates based on risk of BC recurrence			
Foroughi, 2023 (57)	Survey Risk of recurrence not defined by study authors; definitions reported by survey participants varied^a^ Data collection period: NR	HR+/HER2-negative eBC N=141	Average g*BRCA*m testing rate, as reported by HCPs^b^ Patients with low risk of recurrence: 42% to 46% Patients with high risk of recurrence: 63% to 69%

^a^ HCPs used several different educational resources to understand risk of disease recurrence (e.g., guidelines, peer-reviewed journals).

^b^ Testing data provided by oncologists (n=94), surgeons (n=97), and nurse and physician assistants (n=58).

BC, breast cancer; BRCA, breast cancer gene; eBC, early breast cancer; gBRCAm, germline BRCA mutation; HCP, healthcare provider; HER2, human epidermal growth factor receptor 2; HR, hormone receptor; NCCN, National Comprehensive Cancer Network; NR, not reported; TNBC, triple-negative breast cancer

*BRCA* testing rates varied according to other clinical and demographic characteristics including, disease stage, patient race/ethnicity, patient age, and insurance status. *BRCA* testing rates tended to be greater in patients with earlier disease stages (0-III) compared with stage IV or metastatic disease across 3 studies (types of HCP who ordered testing not provided) (37, 39, 42); however, another study reported that physicians routinely ordered g*BRCA*m tests more often for patients with mBC (41%) compared to eBC (23%) (46). *BRCA* testing rates were up to 22% lower among Black patients compared with White patients in 3 studies, including a survey of women diagnosed with eBC from 2007 to 2009, a retrospective cohort study evaluating women diagnosed with BC from 2010 to 2013, and a retrospective database analysis evaluating women diagnosed with TNBC at ≤60 years of age from 2015 to 2020 (39, 52, 58). Younger age was significantly associated with increased rates of *BRCA* testing in 5 studies (data collection dates ranged from 2005 to 2020) (36–39, 43), and private or commercial insurance (compared with Medicare or Medicaid) was significantly associated with increased rates of *BRCA* testing in 2 studies (data collection dates ranged from 2010 to 2020) (37, 39).

#### 3.1.2 Care settings for *BRCA* testing

Three studies reported differences in testing rates when comparing between types of care settings and HCP years of experience (39, 47, 53). A retrospective analysis of the Massachusetts Cancer Registry from 2010 to 2014 reported higher *BRCA* testing rates among women with BC who were treated by physicians affiliated with an academic medical center (58%) compared with other teaching hospitals (48%) or community hospitals (50%; *p* < 0.001) (39). Testing rates were comparable between academic/non-academic settings for patients with TNBC in a chart review study (2015 to 2017); however, the study authors noted that their results may have been biased by exclusion of patients enrolled in clinical trials (47). A medical chart review of community oncology practices (2013 to 2017) found that lower *BRCA* testing rates were observed among solo practitioners or small community practices (1 to 5 physicians) compared to larger practices (53). This study also found lower testing rates were also reported among physicians who treated 50 or fewer patients compared to those who treated more than 50 patients with BC per year, and among physicians who had been practicing for 21 years or more compared to physicians with fewer years of clinical experience (53). Another survey study, which included physicians in both community and academic settings, also found that treating a greater proportion of patients with BC was associated with increased *BRCA* testing; however, in this study, increasing frequency of *BRCA* testing rates was associated with increasing physician age (odds ratio of 1.03 for each 1-year increase in physician age; *p* = 0.02) (59).

Evidence from 2 studies suggests that surgeons most commonly ordered *BRCA* testing for patients, followed by medical oncologists and genetic counselors (31, 60), whereas another study found no significant difference in frequency of ordering between medical oncologists and surgeons (59). Seventy-three percent of surgeons who had seen more than 50 patients with BC in the prior year were confident in discussing genetic testing with their patients, whereas only 35% of surgeons who had seen 1 to 20 patients were confident (31). Furthermore, 37% of surgeons who had seen more than 50 patients in the past year reported that they ordered genetic testing without referring the patient to a genetic counselor compared to 26% of surgeons who had seen 1 to 20 patients with BC (31). Similarly, in another study, *BRCA* testing was ordered most often by surgeons compared to genetic counselors and medical oncologists; however, testing was more likely to be ordered by genetic counselors when surgeons were in academic practice settings (60). The majority (88%) of surgeons reported being confident in their ability to provide appropriate counseling to patients both pre- and post-*BRCA* testing, and approximately half of surgeons reported that this was their standard practice (60). In 1 survey of medical oncologists and surgeons, the frequency of ordering *BRCA* testing did not differ significantly by specialty (surgery versus oncology) or if the physician had access to genetic counseling services when necessary (59).

#### 3.1.3 Timing of *BRCA* testing

According to 3 studies that evaluated surgically treated patients with eBC who received *BRCA* testing, the proportion of patients who received testing before surgery varied widely (14% to 78%) (31, 50, 61). Patient factors associated with increased odds of undergoing g*BRCA*m testing and receiving results before surgery included age <50, college education, stage II disease, TNBC, and greater involvement in surgical decision making (61). Among *BRCA*-tested patients (from studies including patients with mixed stage or advanced BC), nearly 80% were tested within 3 months of diagnosis (44, 46). According to a retrospective, single center study, the rate of g*BRCA*m testing within 3 months of diagnosis rose from 6.9% in 2000 to 2004 to 52.2% of patients in 2010 to 2014 (41). In a retrospective database study, the rates of testing within 6 months of surgery for patients with newly diagnosed invasive BC also increased significantly from 2015 (38%) to 2019 (47%; p<0.001), with a reported absolute percentage increase in testing of 12% among women aged >50 years (p value not reported) (36).

### 3.2 Beliefs and challenges related to *BRCA* testing

#### 3.2.1 Patients

Lower testing rates were observed among patients who reported negative attitudes or a lack of belief in the benefit of testing, according to 3 survey studies (55, 62, 63). Conversely, patients with more positive attitudes about testing and greater agreement regarding the benefits of testing had greater odds of undergoing *BRCA* testing (55). In a survey conducted from 2007 to 2010, patients who did not undergo *BRCA* testing compared with those who did were significantly less likely to believe that *BRCA* testing has benefits to family members (54% vs. 81%), helps to manage their cancer risk (49% vs. 76%), or helps their doctor manage their cancer risk (50% vs. 81%) (55). Approximately 30% of patients with BC believed that testing was too expensive for them to afford (55, 58), and this view was associated with significantly lower odds of testing (55). Furthermore, Black patients were significantly more likely to report negative attitudes regarding *BRCA* testing than White patients (58).

**Figure 2:**
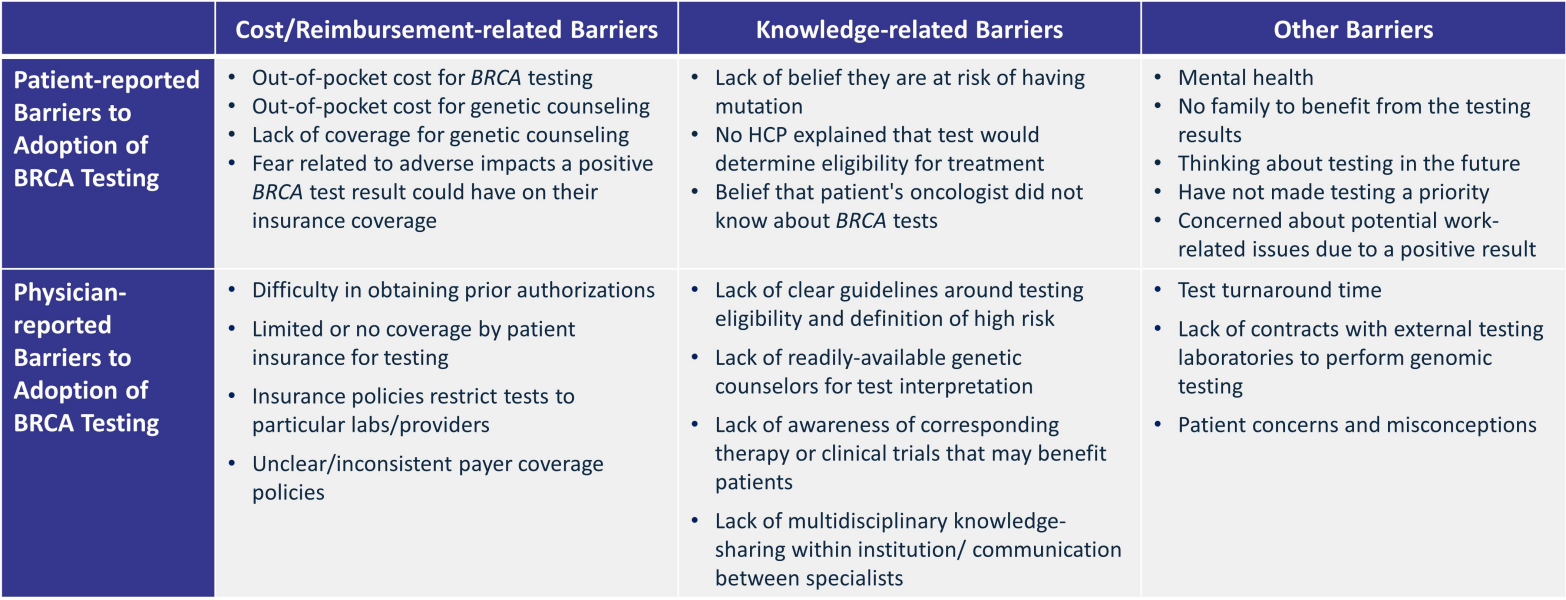
Common patient-reported and physician-reported barriers to *BRCA* testing. *BRCA*, breast cancer gene; HCP, healthcare professional. Source: Based on Beitsch et al., 2014 (60), Earla et al., 2024 (65), Effange et al., 2024 (46), Foroughi et al., 2023 (57), Robles-Rodriguez et al., 2024 (62), Rosenberg et al., 2016 (44), and Szamreta et al., 2022 (66).

Patient-reported drivers of testing included the desire to inform treatment decisions and future health decisions, and the ability to provide benefit to family members (63, 64). Patient-reported barriers to testing were related to costs, mental health concerns, fear related to loss of insurance and impact on family members, and lack of knowledge regarding the benefits of testing or implications for targeted therapy (**Figure 2**) (44, 57, 62, 65). Patients who were diagnosed with HR+/HER2-negative eBC reported reasons provided by their clinicians for why they did not undergo g*BRCA*m testing in a survey published in 2023 (57). These patient-reported reasons included hearing from their clinicians that their level of BC recurrence risk didn’t require g*BRCA*m testing (46%), their sub-type of BC didn’t require testing (30%), their insurance likely wouldn’t cover testing (14%), and they didn’t need g*BRCA*m testing because of their ethnicity/race (5%) (57). Study authors did not provide further analysis of the validity of these patient-reported reasons (57). Notably, 80% of patients who indicated that their clinician said their individual level of risk did not require testing self-reported that they were considered high risk for disease recurrence and should have been tested (57). The study authors did not provide additional details on patients who reported ethnicity and race as a clinician-provided reason for declining *BRCA* testing (57); however, the NCCN Guidelines do not list any ethnicity or race that precludes patients from being tested (18).

#### 3.2.2 Healthcare providers

HCPs reported that testing was logistically challenging and expensive (57, 59). According to HCPs who participated in a 2023 online survey (94 medical oncologists, 97 surgeons, and 58 nurses and physician assistants), payer controls related to g*BRCA*m testing for patients with eBC (including prior authorization and outright rejections) are excessive and burdensome, which may lead to reduced motivation to test patients (57). A survey conducted from 2011 to 2014 of medical oncologists and surgeons reported that 59% of respondents believed *BRCA* testing was too expensive and 15% believed *BRCA* testing was too difficult to arrange (59). Attitudes toward innovation in genomic testing were evaluated using a 5-point Likert scale to measure levels of agreement or disagreement about statements related to use of genetic tests (59). Significantly higher innovation scores were observed among medical oncologists compared with surgeons (*p* < 0.001) and significantly lower innovation scores were observed among providers with little access to genetic counseling (*p* = 0.05) or a larger proportion of uninsured patients (*p* = 0.02) (59). Overall, physicians with a higher innovation score were more likely to order *BRCA* testing (odds ratio: 1.14; *p* = 0.001) (59).

Drivers of *BRCA* testing, as reported in a 2023 survey by genetic counselors and community and academic oncologists who treat patients with eBC, included updated testing guidelines (e.g., NCCN Guidelines), lower costs, closer collaborations between physicians and genetic counselors, and greater patient awareness (65). Common barriers reported by HCPs were related to coverage/reimbursement limitations, lack of multidisciplinary knowledge-sharing or awareness of therapies that may benefit patients, lack of genetic counselors, and patient concerns (**Figure 2**) (46, 57, 60, 65, 66). Results from a 2023 survey of oncology providers (N = 95) found that challenges and barriers to g*BRCA*m testing in BC included patient concerns (47%), access to a genetic counselor (42%), test turnaround time (36%), and reimbursement (28%) (46). According to another 2023 interview-based qualitative study of genetic counselors (n=8) and community and academic oncologists (n=12) who treat patients with eBC, Medicare restricts reimbursement for testing and does not reimburse for genetic counseling sessions (64). Furthermore, HCPs reported that the following patient-related concerns and misconceptions were barriers to testing: costs related to testing were unaffordable, testing will impact insurance coverage, and anxiety (64).

#### 3.2.3 Payers

We identified 1 survey study that evaluated payer-reported beliefs and barriers to g*BRCA*m testing among payers (N = 40) who had a role in medical policy and reimbursement decisions for oncology diagnostic tests (57). Payers reported the belief that physicians overutilize g*BRCA*m testing in patients who are not at high risk of recurrence and therefore not clinically indicated for testing (57). The top 5 payer-reported barriers to g*BRCA*m testing included lack of complete documentation for reimbursement, overutilization of testing by clinicians, lack of clear national guidelines around eligibility, lack of utility of g*BRCA*m testing for treatment selection, and lack of internal expertise around *BRCA* testing (57).

## 4 Discussion

Real-world *BRCA* testing trends observed in the literature indicate that *BRCA* testing rates have increased since the early 2000’s (36, 37, 39–41, 43–45). Higher real-world *BRCA* testing rates were observed among patients with a high risk of carrying a *BRCA* mutation and patients with a high risk of disease recurrence, compared to patients with low or moderate risk (51, 55, 57, 59). However, not all high risk patients receive testing; for example, HCPs reported that only approximately two-thirds of patients with HR+/HER2- eBC who were at high risk of recurrence received *gBRCA*m testing (57). These results suggest HCPs may be less likely to order testing in the HR+ population despite eligibility for *gBRCA*m testing based on risk criteria.

Evidence showed that patients, physicians, and payers all report that a lack of clear guidelines around *BRCA* testing eligibility is a barrier to testing (57, 66), which could be related to variability in interpretation or implementation of evolving guidelines. Furthermore, lower testing rates observed among physicians in small community settings or among physicians who have less positive attitudes toward innovation in genomic testing underscores the need for continued education to update physicians on the latest treatment advances and associated guideline changes, such as the approval of olaparib in 2022 as an option for patients with g*BRCA*m, HER2-negative eBC at high risk of disease recurrence (24, 53, 59). Although there has been an increase in the uptake of *BRCA* testing over time, the impact of olaparib’s approval on *BRCA* testing rates was difficult to assess in this review, as none of the identified studies evaluating eBC had a data collection period of 2023 or later. The impact of olaparib on testing rates will be important to evaluate in future research studies to help inform strategies to increase the uptake of *BRCA* testing.

Although most patients with BC were aware of *BRCA* testing, knowledge gaps regarding the benefits of testing and implications for targeted therapy were observed (38, 62). A contributing factor to patients’ lack of understanding around the usefulness of *BRCA* testing may be related to the increased use of multiple gene panels that test for *BRCA* in addition to other pathogenic gene variants, compared with *BRCA*-only tests (40). Further research to explore patients’ awareness and knowledge of panel testing compared to *BRCA*-only testing is warranted.

Variable access to genetic counseling was reported by physicians as both a driver and a barrier to *BRCA* testing (46, 65, 66). Some physicians may lack the expertise or confidence to provide appropriate counseling before and after *BRCA* testing, suggesting an opportunity for clinician education (31, 60). Approximately half of patients reported discussing their testing results with a genetic counselor (higher risk of carrying a g*BRCA*m: 57%; average risk of carrying a g*BRCA*m: 42%) (31). Importantly, the use of a genetic counselor has been associated with significant increases in *BRCA* testing referral rates among patients with BC who had a personal or family history-based indication for testing (56). This suggests that increasing the availability of genetic counselors could aid in increasing the uptake of *BRCA* testing, particularly among patients with a high risk of carrying a *BRCA* mutation.

Costs and lack of insurance coverage were frequently cited by both patients and HCPs as barriers to *BRCA* testing (44, 55, 57, 66). Although the cost of testing has decreased over time (30, 31), HCPs and patients may be unaware of the availability of low or no cost testing through various channels (67), highlighting an additional opportunity for education initiatives.

### 4.1 Limitations

This literature review identified evidence gaps and associated limitations. Firstly, most of the identified studies (21 of 34) had data collection periods that occurred prior to 2020. This represents a significant limitation, given the rapid evolution of personalized medicine (e.g., testing guidelines updates, availability of multigene panels, and the recent approval of olaparib for treating HER2- high-risk eBC). Therefore, our results may not reflect recent testing rates within the past 5 years and provide limited insight into the most recent gaps in g*BRCA*m testing. In addition, our study included published data that reported results specifically for *BRCA* testing and excluded studies evaluating genetic testing in general and/or panel testing (which may have included *BRCA* testing). Given that sequential testing has higher results turnaround times and cost (68) and that patients may be eligible for genetic testing other than g*BRCA*m, the impact of using multigene panel testing versus standalone *BRCA* testing on testing rates and perceived barriers should be investigated. Furthermore, we did not evaluate cascade testing and its impact on *BRCA* testing rates among family members of patients with BC or on the proportion of patients who received *BRCA* testing prior to their BC diagnosis. Another limitation of the evidence identified in this review was the substantial heterogeneity across study designs and populations assessed. Furthermore, not all studies clarified if testing referred to *gBRCA*m and/or somatic *BRCA* testing. This heterogeneity limits the ability to compare results across studies and highlights the need for high-quality studies that compare g*BRCA*m testing uptake across populations. Lastly, we only identified 1 study evaluating the payer perspective, demonstrating a need for further research on payer controls for *BRCA* testing and payer misconceptions. Given these limitations, further evaluation of more recent real-world *BRCA* testing trends and beliefs/barriers related to *BRCA* testing in the US is needed to reflect current practices, the impact of olaparib’s approval in 2022 in g*BRCA*m eBC, and updated guidelines.

### 4.2 Opportunities for improvement in *BRCA* testing

Findings from our review reveal several opportunities for improvement in *BRCA* testing in BC, particularly among patients with HR+/HER2-negative eBC, including:

- Educating HCPs (particularly those in community settings), payers, and patients is needed, especially considering the evolving guidelines and the complex therapeutic landscape, which may be facilitated by increasing awareness of the availability of simple guidelines [e.g., ASBrS (20), ASCO-SSO (19)]. Specifically, educating HCPs and payers on testing eligibility, given the evolving definition of risk of carrying the *BRCA* mutation according to guidelines. Since the presence of a *BRCA* mutation has historically been considered prognostic, there is an opportunity in the eBC setting to educate patients and providers on OlympiA trial data. Given the greater prevalence of negative attitudes towards *BRCA* testing among Black patients compared to White patients, further education for Black patients is warranted to ensure that patients are aware of how receiving *BRCA* testing may result in opportunities for targeted therapy.
- Improving workflows to increase access to testing results and/or enable the flow of information for both HCPs and patients may alleviate the logistical difficulties associated with *BRCA* testing and associated delays in care. During the initial visit following a BC diagnosis, patients may be overwhelmed by the amount of information they have received, and HCPs have limited time to spend with each patient. Improved workflows could prompt HCPs to order tests and discuss recommendations for testing with patients. Additional reminders that prompt HCPs to follow-up with patients to complete testing may also be useful.
- Innovative models of care could help to address bottlenecks in access to genetic counselors and potentially reduce burden for patients. For example, telehealth appointments, virtual education, and at-home testing could significantly reduce travel burden for patients while simultaneously helping them feel more comfortable with the testing process. Increased use of collaborative care teams, including genetic counselors, can also help reduce the burden on physicians who have limited time to devote to discussing genetic-related information.
- Information on cost and accessibility should be provided for reimbursement policies and on the long-term financial benefits of testing to help overcome patient and HCP perceptions of coverage and cost barriers. Additionally, an up-to-date resource providing HCPs with available testing kits and relevant considerations (e.g., care settings) could help with genetic test decision making.
- Engaging patient advocacy groups (including community-specific organizations) as a strategy to help reach and educate patients who have less trust in the medical system should also be considered as an avenue for increasing uptake of *BRCA* testing among eligible patients.
