# Supplementary figures and images for: BRCA testing patterns in breast cancer over time in the United States: challenges and opportunities for improvement
Source: Front Oncol. 2026 Apr 27;16:1797497. doi: 10.3389/fonc.2026.1797497 (PMC13158077; doi:10.3389/fonc.2026.1797497)

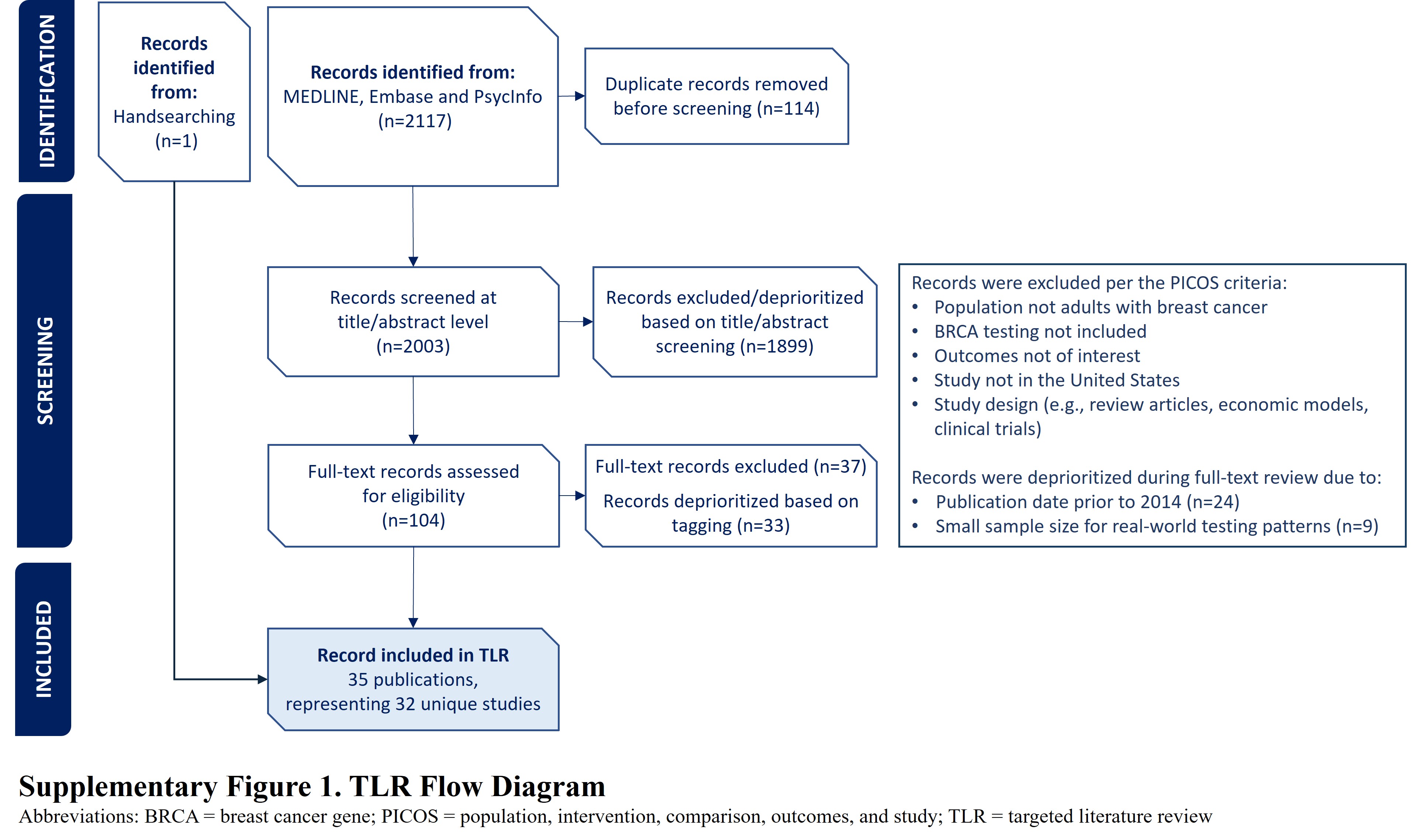

Supplement: Supplementary file 1 [file Image1.jpeg]
