# Supplementary material for: BRCA testing patterns in breast cancer over time in the United States: challenges and opportunities for improvement
Source: Front Oncol. 2026 Apr 27;16:1797497. doi: 10.3389/fonc.2026.1797497 (PMC13158077; doi:10.3389/fonc.2026.1797497)
Supplement: Supplementary file 3 [file Table2.docx]

**Supplementary Table 2. OVID Algorithm – PsycInfo**

| **Search Number** | **Search Terms** | **Results** |
| --- | --- | --- |
| 1 | (BRCA$ or gBRCA or sBRCA).ti,ab. | 653 |
| 2 | (medicare or medicaid or african american$ or hispanic american$ or arab american$ or Appalachia$ or Great Lake$ or Mid?atlantic or Mid?west$ or new england or pacific state$).ti,ab. | 84227 |
| 3 | (united states or usa or “u.s.a.” or “u.s.” or veteran$ or SEER or alabama or montgomery or Alaska$ or juneau or anchorage or arizona or phoenix or arkansas or little rock or California$ or sacramento or los angeles or colorado or denver or connecticut or hartford or bridgeport or delaware or dover or wilmington or florida or tallahassee or jacksonville or miami or atlanta or hawai$ or honolulu or idaho or boise or illinois or springfield or chicago or indiana or indianapolis or iowa or des moines or kansas or topeka or wichita or kentucky or frankfort or louisville or louisiana or baton rouge or new orleans or maine or augusta or portland or maryland or annapolis or baltimore or massachusetts or boston or michigan or lansing or detroit or minnesota or “st. paul” or minneapolis or mississippi or jackson or missouri or jefferson city or montana or billings or nebraska or omaha or nevada or carson city or las vegas or new hampshire or concord or new jersey or trenton or newark or new Mexico or santa fe or albuquerque or new york or albany or north carolina or raleigh or north dakota or bismarck or fargo or ohio or columbus or oklahoma or oregon or salem or pennsylvania or harrisburg or philadelphia or rhode island or providence or south carolina or columbia or charleston or south dakota or sioux falls or tennessee or nashville or texas or austin or houston or utah or salt lake city or vermont or montpelier or burlington or virginia or richmond or washington or olympia or seattle or wisconsin or madison or milwaukee or wyoming or Cheyenne or Charlotte or Cleveland or Dallas or Denver or Detroit or District of Columbia or El Paso or Fort Worth or Fresno or Long Beach or Memphis or Mesa or Oakland or San Antonio or San Diego or San Francisco or San Jose or San Juan or Seattle or Tucson or Tulsa).ti,ab. | 605139 |
| **4** | **1 and (2 or 3)** | **89** |
