# Supplementary material for: BRCA testing patterns in breast cancer over time in the United States: challenges and opportunities for improvement
Source: Front Oncol. 2026 Apr 27;16:1797497. doi: 10.3389/fonc.2026.1797497 (PMC13158077; doi:10.3389/fonc.2026.1797497)
Supplement: Supplementary file 4 [file Table3.docx]

**Supplementary Table 3. PICOS Criteria for Study Inclusion**

| **Criteria** | **Inclusion** | **Exclusion** |
| --- | --- | --- |
| **Population** | - Women and men with BC (any stage) who are eligible for BRCA testing - Eligibility for BRCA testing will be based on study definition if available - Healthcare professionals who decide/recommend/administer BRCA testing, including but not limited to: - Oncologists - Pathologists - Surgeons - Genetic medicine specialists and genetic counselors who advise patients on BRCA testing - Nurses - Healthcare providers and payers who decide about/recommend BRCA testing | Women and men who are eligible for BRCA testing but have not been diagnosed with cancer |
| **Interventions** | *BRCA* testing (*BRCA1*, *BRCA2*, germline *BRCA*, any type) | Not applicable |
| **Comparators** | Any or none | Not applicable |
| **Outcomes** | - RW BRCA testing patterns, including: - Proportion of patients with breast cancer receiving the BRCA test - Proportion of patients with breast cancer refusing the BRCA test - Timing of the *BRCA* test - Misconceptions (e.g., beliefs regarding testing guidelines and procedures that are not supported by current evidence), barriers, beliefs, or attitudes about BRCA testing for treatment decision, as reported by patients or healthcare professionals | Clinical outcomes, economic outcomes, quality of life, PROs |
| **Study Design** | - Observational studies - Qualitative studies (e.g., interviews, focus groups) - Surveys | - Clinical trials - Case reports and case series - Systematic or narrative reviews^a^ - Comments/commentary - Editorials - Animal studies - Cytology, in vitro/in vivo studies, gene expression/protein expression studies |
| **Other** | - **Geography**: US only - **Language**: No language limit - **Publication date**: - No limit on publication date for articles - Conference proceedings were limited from 2022 to 2024 | - Non-US - Conference abstracts prior to 2022 |

^a^ Reviews were excluded from the SLR, but the bibliographies of recently published SLRs were cross checked for any publications that might have not been captured by the searches, as a quality assurance method.

Abbreviations: BC = breast cancer; BRCA = breast cancer gene; PICOS = population, interventions and comparisons, outcomes, and study design; PRO = patient-reported outcomes; US = United States
